# Hydrological Regime and Anthropogenic Pressure Shape the Population Structure of *Lycium dasystemum* in Tugai Forests of the Syr Darya Basin

**DOI:** 10.3390/plants15142168

**Published:** 2026-07-14

**Authors:** Shakhnoza Saribaeva, Khabibullo Shomurodov, Bekhzod Adilov, Bekhruz Khabibullaev, Tashkhanim Rakhimova, Nodira Rakhimova, Vasila Sharipova, Zhasur Sadinov, Azamat Sultamuratov, Farrukh Polvonov, Allabek Mirzambetov, Andrey Korolyuk, Giuseppe Fenu

**Affiliations:** 1 Institute of Botany Academy of Sciences Republic of Uzbekistan, Tashkent 100125, Uzbekistan; shakhnozasaribaeva@gmail.com (S.S.); h.shomurodov@mail.ru (K.S.); bekhzod_a@mail.ru (B.A.); bekh.xabibullaev@mail.ru (B.K.); tashkhanim@mail.ru (T.R.); rakhimovanodi@mail.ru (N.R.); vasila_82@mail.ru (V.S.); jasursadinov5405@gmail.com (Z.S.); as.azamatsultamuratov@gmail.com (A.S.); f.polvonov@bk.ru (F.P.); mirzambetovallabek@gmail.com (A.M.); 2 Central Siberian Botanical Garden, Siberian Branch, Russian Academy of Sciences, Novosibirsk 630090, Russia; akorolyuk@rambler.ru; 3 Department of Life and Environmental Sciences, University of Cagliari, 09123 Cagliari, Italy

**Keywords:** Tugai forests, population structure, floodplain ecosystems, hydrological alteration, population stability, Central Asia

## Abstract

Tugai forests represent key riparian ecosystems of the arid landscapes of Central Asia, where vegetation dynamics are strongly influenced by seasonal hydrological variability and increasing anthropogenic pressure. Assessing the population structure of dominant shrub species can provide important insights into the condition and resilience of these floodplain ecosystems. This study examines the demographic structure, vitality, and morphological variability of *Lycium dasystemum* populations in the Syr Darya River valley. Field surveys were conducted in six subpopulations located in Tugai forests differing in hydrological conditions and levels of anthropogenic disturbance. Population structure was assessed using ontogenetic spectra, ecological density, and regeneration indices, while vitality indices and morphological traits were analyzed to evaluate population condition and phenotypic variability. Species turnover dominated β-diversity patterns (βsim up to 81%), indicating floristic differentiation among the studied communities. Clear spatial differences in demographic parameters were detected among subpopulations. Subpopulations occurring in relatively stable habitats showed more balanced ontogenetic spectra, higher vitality indices, and signs of active regeneration, whereas subpopulations exposed to stronger anthropogenic pressure were characterized by demographic imbalance, reduced recruitment, and lower ecological density. Morphological analyses revealed moderate phenotypic plasticity, suggesting that some traits may be associated with local floodplain habitat conditions. These findings indicate that maintaining suitable floodplain moisture conditions and regulating grazing pressure may contribute to the persistence of *L. dasystemum* populations and the conservation of Tugai vegetation in the Syr Darya basin.

## 1. Introduction

Tugai forests represent unique floodplain ecosystems of the arid and semi-arid regions of Central Asia. These riparian communities play a key role in maintaining ecosystem stability by supporting biodiversity, stabilizing soils, and regulating hydrological processes, while also functioning as ecological corridors along river valleys [1,2]. However, during recent decades, these ecosystems have experienced substantial degradation due to alterations of river hydrological regimes, increasing anthropogenic pressure, and ongoing climate change [3,4].

In ecological theory, the dynamics of floodplain ecosystems are commonly interpreted within the framework of the Flood Pulse Concept, which proposes that periodic flooding generates spatial habitat heterogeneity, regulates resource distribution, and supports the regeneration of riparian vegetation [5,6]. Alterations of natural hydrological regimes are widely recognized as a major driver of change in riparian plant communities and river ecosystems [7,8,9,10]. In regulated river systems, where hydrological disturbances are modified by dams, irrigation infrastructure, and other human activities, the natural disturbance regime becomes disrupted, leading to restructuring of plant communities and shifts in population demographic characteristics [11,12,13].

The Syr Darya River is one of the largest river systems in Central Asia and represents a major hydrological axis of the region. It is formed by the confluence of the Naryn and Karadarya rivers in the eastern Fergana Valley and flows across several countries before discharging into the Aral Sea basin. The drainage basin covers approximately 219,000 km^2^ and contains extensive floodplains that historically supported diverse Tugai vegetation. However, large-scale regulation of river discharge through reservoir construction, irrigation development, and water diversion has substantially altered the natural hydrological regime of the Syr Darya basin [14,15]. As a consequence, seasonal flood pulses that previously played a key role in groundwater recharge, nutrient accumulation, and regeneration of floodplain vegetation have become less frequent and less intense.

Hydrological transformations are further accompanied by increasing anthropogenic pressure within river valleys. Intensive land use, including livestock grazing, agricultural expansion, and wood harvesting, accelerates the degradation of Tugai ecosystems. These processes often lead to soil compaction, reduction in phytomass, simplification of plant community structure and loss of taxonomic diversity. Understanding how hydrological changes and anthropogenic disturbances affect the structure and stability of Tugai vegetation has therefore become an important ecological and conservation challenge for Central Asia.

In recent years, studies of Tugai forests in Central Asia have expanded and increasingly address issues of biodiversity, ecosystem functioning, and anthropogenic transformation [16,17,18,19,20,21,22,23]. Research conducted in the lower Amudarya basin has shown that increasing anthropogenic pressure can substantially alter vegetation structure, including an increased dominance of ruderal annual plants [24]. Other studies highlight the strong dependence of tugai vegetation dynamics on hydrological conditions, particularly in communities dominated by species of the genera *Elaeagnus*, *Tamarix*, and *Populus* [25,26].

Despite the growing body of research, population-level studies of individual species within Tugai ecosystems remain limited, particularly in Uzbekistan. Most existing studies focus on rare and endangered plant species included in the Red Data Book of Uzbekistan [27], whereas the demographic stability and adaptive responses of characteristic Tugai species under changing hydrological regimes and increasing anthropogenic pressure remain poorly understood [28]. However, analyses of population structure and vitality are essential for evaluating the resilience of plant communities and the functioning of floodplain ecosystems.

The genus *Lycium* L. (Solanaceae) is an important component of Tugai vegetation. Species of this genus are widely distributed in arid and semi-arid regions and play a significant role in the structure of shrub communities in floodplain landscapes [24,29,30,31]. Beyond their ecological role, many *Lycium* species are widely used for food and medicinal purposes. Their fruits, commonly known as goji berries, have attracted considerable attention due to their nutritional and pharmacological properties [32,33,34,35]. Phytochemical studies show that *Lycium* species contain numerous bioactive compounds, including polysaccharides, carotenoids, flavonoids, and alkaloids [36].

*Lycium dasystemum* Pojark. is a characteristic shrub of Tugai ecosystems in the Syr Darya River basin. In addition to its potential medicinal value, this species plays an important structural and functional role within floodplain plant communities by contributing to the formation of the shrub layer, providing microhabitats for associated herbaceous species, and supporting habitat heterogeneity in riparian landscapes. As a spiny shrub, it may also reduce local grazing pressure on understory vegetation and contribute to the stabilization of disturbed floodplain soils. Therefore, changes in its population structure and vitality may reflect broader alterations in the ecological condition of Tugai habitats. However, its populations may be sensitive to alterations of hydrological regimes and increasing anthropogenic pressure, which can influence regeneration processes, population structure, and long-term population stability.

The main aim of this study was to assess the current state of *L. dasystemum* populations in the upper and middle reaches of the Syr Darya River. We analyzed population structure, vitality, and morphological variability under both different hydrological conditions and levels of anthropogenic disturbance. This study provides the first integrated analysis of demographic structure, vitality characteristics, and morphological variability of *L. dasystemum* populations in Syr Darya tugai ecosystems under varying hydrological regimes and anthropogenic pressure. By linking hydrological dynamics with population-level responses, this study aims to improve understanding of species resilience in degraded tugai ecosystems and provide a scientific basis for conservation and sustainable management of floodplain forests in Central Asia.

Based on these objectives, the following hypotheses were tested: (i) Hydrological conditions represent a key factor determining the structure and demographic patterns of *L. dasystemum* populations in the Syr Darya floodplain. Populations under more stable hydrological conditions are expected to exhibit balanced ontogenetic spectra and higher regeneration levels. (ii) Anthropogenic disturbance significantly influences demographic stability; under increased disturbance, populations are expected to show demographic imbalance, reduced proportions of pre-generative individuals, and lower ecological density. (iii) Morphological variability reflects adaptive responses to environmental gradients, and morphometric traits of *L. dasystemum* are expected to vary along gradients of hydrological conditions and anthropogenic disturbance.

## 2. Materials and Methods

### 2.1. Study Species and Study Area

**Figure 1:**
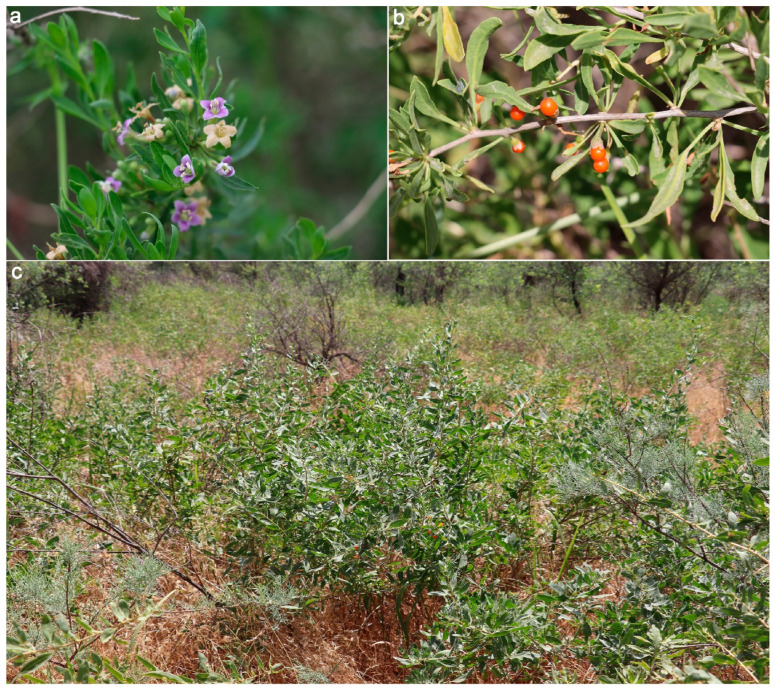
Photo of *Lycium dasystemum*: (**a**) flowers, (**b**) fruits, (**c**) background.

The study focused on *L. dasystemum* Pojark. (Solanaceae; Figure 1), a branched spiny shrub reaching up to 1.5 m in height. The species is distributed across Afghanistan, Central Asia, China, and Iran [37]. Within Uzbekistan, *L. dasystemum* is primarily associated with Tugai forests and riparian shrub communities occurring along major river valleys [38].

The Syr Darya River is one of the largest transboundary rivers in Central Asia, flowing through Kyrgyzstan, Tajikistan, Uzbekistan, and Kazakhstan before discharging into the Aral Sea basin. The river plays a crucial role in the formation of floodplain and Tugai ecosystems and represents a major water resource for agriculture and human populations in the region [2,14,39,40].

**Figure 2:**
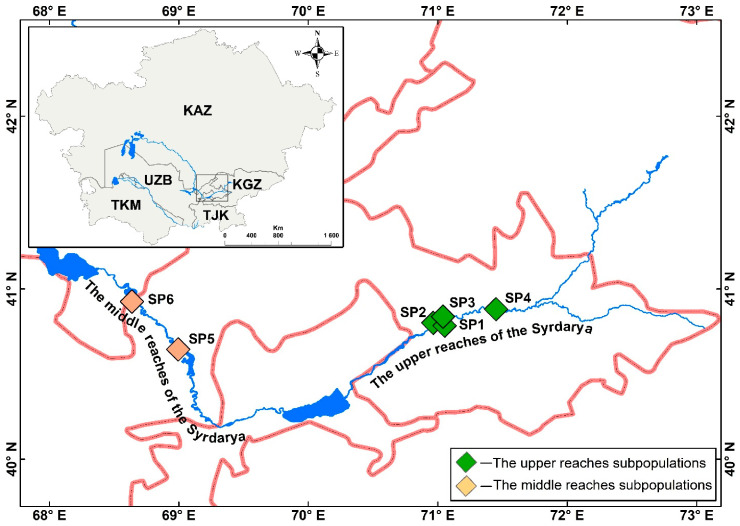
Study site of the *Lycium dasystemum* subpopulations along the Syrdarya River basin. The blue lines and areas represent rivers and water bodies, respectively, while the red lines indicate administrative boundaries. Green and orange diamonds represent sampling sites of the upper and middle reaches subpopulations, respectively.

In this study, subpopulations of *L. dasystemum* located in the upper and middle reaches of the Syr Darya River within Uzbekistan were investigated. The study area covered approximately 250 km of the river corridor. The lower reaches of the river, located within Kazakhstan, were not included in the analysis (Figure 2).

### 2.2. Hydrological Data and Surface Water Dynamics

Hydrological data were obtained from gauging stations located in the upper and middle reaches of the Syr Darya River for the period 2020–2024. Monthly discharge records were used in the analysis. These data were obtained from regional hydrometeorological monitoring services operating within the Syr Darya basin.

When several gauging stations were located within the same river section (upper or middle reach), monthly mean values were calculated to represent the hydrological conditions of that river segment. Because site-specific hydrological measurements were not available for each subpopulation, the hydrological data were used to characterize the general seasonal and interannual background of the upper and middle reaches of the Syr Darya River. These data were not treated as direct explanatory variables for differences among individual subpopulations.

### 2.3. Field Sampling and Study Design

Four subpopulations of *L. dasystemum* were identified in the upper Syr Darya valley and an additional two in the middle reach (Figure 2). For each subpopulation, geographic coordinates, habitat characteristics, dominant plant species, and the level of anthropogenic disturbance were recorded (see Appendix A, Table A1).

Geobotanical surveys were conducted during the growing seasons of 2020–2024 on 100 m^2^ plots established within each subpopulation, following the Braun–Blanquet method [41]. Plots were positioned in the most representative parts of the subpopulations to capture the dominant vegetation structure. Within each plot, species composition, total vegetation cover, species abundance, dominant taxa, and the structural role of *L. dasystemum* were recorded. These data were used to analyse floristic diversity, community structure, and the contribution of the target species to plant communities.

Subpopulation age structure was assessed within the same monitoring plots. Within each 100 m^2^ plot, a 20 m^2^ subplot was established in the most representative part of the subpopulation. All individuals within this area were counted.

Individuals were assigned to seven ontogenetic stages: juvenile (j), immature (im), virginile (v), young generative (g1), mature generative (g2), old generative (g3), and senile (s). Ontogenetic classification was based on morphological criteria following the methodological framework of Uranov (1975) and Zaugolnova (1994) [42,43]. Diagnostic traits included the development of vegetative and reproductive organs, branching structure, shoot and leaf size, and visible signs of ageing.

To refine diagnostic criteria, ten representative individuals from each subpopulation were collected as herbarium specimens. Ontogenetic spectra were constructed based on the distribution of individuals among age classes to analyze the demographic structure of the subpopulations.

Morphological variability and vitality were assessed using ten mature generative individuals (g2) from each subpopulation. For each plant, key biometric parameters were measured and used to calculate mean values, coefficients of variation, and indices of phytocoenotic plasticity.

### 2.4. Assessment of Anthropogenic Disturbance

Anthropogenic disturbance was evaluated in each subpopulation using a five-stage degradation scale (I–V) according to the method of A.B. Kabanov (2007) [44]. The assessment was based on the proportion of trampled area and the degree of transformation of the vegetation cover.

The disturbance stages were defined as follows:

Stage I (undisturbed): <1% of the area trampled; vegetation cover largely intact and regeneration viable.

Stage II: 1.1–5% trampled; partial disturbance of the litter layer and formation of footpaths.

Stage III: 5.1–10% trampled; pronounced litter disturbance, exposure of mineral soil horizons, and soil compaction.

Stage IV: 10.1–25% trampled; substantial vegetation degradation and widespread trampling with signs of dieback in woody plants.

Stage V (severe degradation): >25% trampled; destruction of ground vegetation and strong soil compaction and/or erosion.

### 2.5. Data Analysis

Floristic differentiation among subpopulations was analyzed using Two-Way Indicator Species Analysis (TWINSPAN version 2.3), a hierarchical divisive classification method based on correspondence analysis and transformation of species into pseudospecies [45,46]. Standard cut levels (0, 2, 5, 10, 20) were used for pseudospecies generation. Classification was based on χ^2^ distances, and eigenvalues were used to evaluate the strength of divisions. The first division was considered ecologically meaningful [45].

Local (α) diversity was evaluated using species richness (S), Shannon diversity index (H′), and Simpson diversity index (1 − D). The Shannon index reflects both species richness and evenness [47], whereas the Simpson index describes dominance patterns [48]. All calculations were performed in R software (version 4.4.3, R Core Team 2026) using the vegan package (version 2.7-5) [49].

Pairwise floristic similarity between subpopulations was calculated using the Sorensen similarity index based on presence–absence data [50]. In R, dissimilarity was calculated using the Bray–Curtis index with binary transformation [51], after which similarity was obtained as

$$S_{sor}=1-D_{sor}$$

Results were expressed as percentages.

Total β-diversity was partitioned into two components: species turnover (βsim) and nestedness (βsne), following the method of Baselga (2010) [52]. The analysis was conducted in R using the betapart package (version 1.6) [53].

Population age structure was analyzed using methods of plant coenopopulation biology [54]. Ontogenetic spectra were defined as the proportional distribution of individuals among age classes. Four types of ontogenetic spectra were distinguished: left-skewed, centered, right-skewed, and bimodal.

To evaluate demographic status, the “delta–omega” approach [42,54] was adopted, where Δ—age index reflecting the ontogenetic level of the population and ω—efficiency index describing the contribution of individuals of different ontogenetic stages to resource utilization (Table 1).

The age index was calculated as

$$\Delta =\frac{\sum (K_{i}\cdot M_{i})}{N}$$

where $K_{i}$—weighting coefficient of the ontogenetic stage, $M_{i}$—density of the stage, N—total population density.

The population efficiency index (ω) was calculated using the following formula:

$$\omega =\frac{\sum (n_{i}\cdot e_{i})}{\sum n_{i}}$$

where $n_{i}$—number of individuals in the *i*th ontogenetic stage; $e_{i}$—efficiency coefficient corresponding to the *i*th ontogenetic stage.

The index characterizes the contribution of individuals at different ontogenetic stages to the overall functional state of the population and allows comparison of demographic structure among subpopulations.

Population density (D) was calculated as

$$D=\frac{\sum N_{i}}{S_{total}}$$

where $N_{i}$—number of individuals, $S_{total}$—total sampling area.

Ecological density was calculated as the number of individuals per unit of space actually available for colonization [55].

Processes of regeneration and generational replacement were assessed using the following indices.

Regeneration index (Iv):

$$I_{v}=\frac{N_{j}+N_{im}+N_{v}}{N_{g1}+N_{g2}+N_{g3}}$$

where $N_{j}$—number of juvenile individuals; $N_{im}$—number of immature individuals; $N_{v}$—number of virginile individuals; $N_{g1}$, $N_{g2}$, $N_{g3}$—numbers of young, mature, and old generative individuals.

Values of Iv close to 0–0.17 indicate a low level of regeneration and are characteristic of ageing populations [56].

Replacement index (I_z_):

$$I_{z}=\frac{N_{j}+N_{im}+N_{v}}{(N_{g1}+N_{g2}+N_{g3})+N_{s}}$$

where $N_{s}$—number of senile individuals.

Values of I_z_ > 1 indicate that the population is capable of effective replacement of older individuals by younger ones.

Ageing index (Is):

$$I_{s}=\frac{N_{s}}{N_{j}+N_{im}+N_{v}}$$

This index reflects the degree of population ageing and characterizes the relative contribution of senile individuals compared with the prereproductive stages.

Vitality of individuals was evaluated using normalized values of morphological traits relative to mean values across all subpopulations. Individuals were assigned to three vitality classes: high (a), medium (b), and low (c) [57].

The vitality type of each subpopulation was determined using the Q criterion:

$$Q=\frac{1}{2}(a+b)$$

Based on the relationship between Q and the proportion of individuals of class c, subpopulations were classified as prosperous (Q > c), equilibrium (Q = c), and depressed (Q < c).

The prosperity index was calculated as

$$IQ=\frac{a+b}{2c}$$

Values of IQ > 1 indicate a prosperous population state, whereas IQ < 1 indicates a depressed state.

Additionally, the population vitality index (IVC) proposed by Ishbirdin and Ishmuratova [58] was applied. The index was calculated on the basis of the size spectra of generative individuals using the following formula:

$$IVC=\frac{\sum_{i=1}^{N}\left(\frac{X_{i}^{1}}{X_{i}^{2}}\right)}{N}$$

where $X_{i}^{1}$—mean value of the i-th morphological trait in a given subpopulation; $X_{i}^{2}$—mean value of the i-th morphological trait calculated across all subpopulations; N—number of analyzed morphological traits.

The IVC reflects the relative correspondence of morphological parameters of individuals to the environmental conditions of their habitat and is used to compare the vitality status of different subpopulations.

Morphological variability of traits was assessed using the coefficient of variation (CV, %). Variability levels were interpreted according to [59]: <7%—very low; 7–12%—low; 13–20%—moderate; 21–40%—high; 40%—very high.

Phytocoenotic plasticity (Ip) was calculated as

$$I_{p}=\frac{A-B}{A}$$

where A—maximum mean value of a trait during the observation period, B—minimum mean value.

Arithmetic means and standard errors (mean ± SE) were calculated for all morphological traits. Prior to applying parametric tests, data were examined for normality and homogeneity of variance. Differences among subpopulations were tested using Student’s *t*-test, and only traits showing statistically significant differences (*p* < 0.05) were included in further analyses (see Appendix A, Table A2).

Hydrological variability was characterized using descriptive statistics including mean values, standard deviation, minimum and maximum values, median, and coefficient of variation (CV).

Seasonal differences in hydrological values were tested using the nonparametric Kruskal–Wallis test applied separately for the upper and middle reaches of the river.

All statistical analyses were performed in the R statistical environment [60] using the packages dplyr (version 1.1.4) [61], trend (version 1.1.6) [62], and forecast (version 8.24.0) [63].

Because each subpopulation was represented by one main sampling plot and the number of subpopulations was limited, relationships between hydrological conditions, anthropogenic disturbance, and population parameters were interpreted descriptively. Therefore, the study does not attempt to infer direct causal effects, and the observed patterns are discussed as associations rather than statistically confirmed causal relationships.

## 3. Results

### 3.1. Long-Term Dynamics of Water Resources in the Syr Darya River

**Figure 3:**
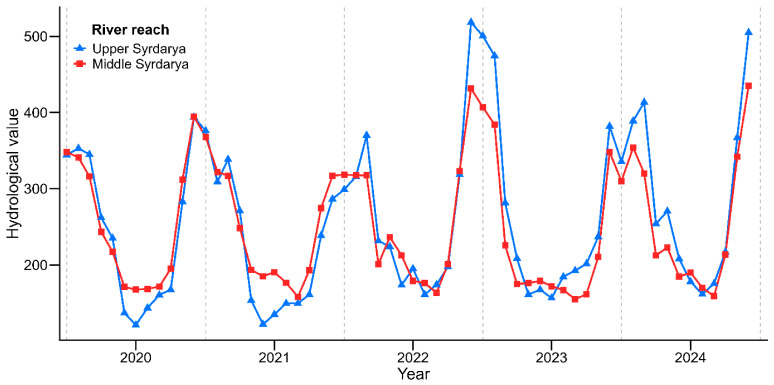
Monthly dynamics of hydrological values in the upper and middle reaches of the Syr Darya River (2019–2024).

River discharge in the Syr Darya basin exhibited a strongly seasonal but interannually stable hydrological regime during the 2020–2024 observation period. In both studied river sections, discharge increased markedly during spring and early summer and declined during late summer and autumn (Figure 3).

Statistical analyses confirmed significant seasonal variation in river discharge. The Kruskal–Wallis test revealed significant differences among months both in the upstream section (χ^2^ = 49.17, df = 11, *p* < 0.001) and in the midstream section (χ^2^ = 50.64, df = 11, *p* < 0.001).

Hydrological dynamics of the upstream and midstream sections were highly synchronized. Pearson correlation analysis revealed a strong positive relationship between discharge values in the two river sections (r = 0.95, *p* < 0.001; see Appendix A, Table A3 and Table A4).

Comparative analysis of interannual variability showed no statistically significant differences among years. The Kruskal–Wallis test indicated no interannual differences in either the upstream section (χ^2^ = 3.58, df = 4, *p* = 0.466) or the midstream section (χ^2^ = 1.85, df = 4, *p* = 0.763). Thus, the hydrological data indicate a pronounced seasonal regime but do not provide evidence of a strong divergence between the upper and middle river reaches during the 2020–2024 period. Therefore, these data were used as background information for interpreting the floodplain context rather than as a direct explanation of differences among the six subpopulations.

### 3.2. Anthropogenic Pressure Across Subpopulations

Assessment of habitat disturbance revealed pronounced spatial heterogeneity in anthropogenic pressure among the six studied subpopulations of *L. dasystemum*. The degree of disturbance varied both along the river corridor and among local sites, creating a mosaic of contrasting ecological conditions.

The most pronounced trampling impact was observed in subpopulation SP3, where the proportion of disturbed surface exceeded 25%, corresponding to stage V of degradation and indicating severe anthropogenic pressure. Subpopulations SP2 and SP5 were characterized by moderately high disturbance levels, with 10–25% of the surface affected, corresponding to stage IV of degradation.

Lower levels of disturbance were recorded in SP1, SP4, and SP6. In SP1, the disturbed surface accounted for 5–10%, corresponding to stage III of degradation. In SP4 and SP6, trampling affected less than 5% of the surface, corresponding to stage II of degradation and indicating relatively weak anthropogenic impact.

Subpopulations located in the upper reach of the river displayed a broader range of disturbance levels, whereas sites in the midstream section were generally characterized by low to moderately high anthropogenic pressure. These differences in disturbance intensity provide an ecological context for interpreting variation in demographic structure, regeneration capacity, ecological density, and vitality parameters of *L. dasystemum* subpopulations.

### 3.3. Alpha Diversity Patterns

Indices of α-diversity varied substantially among the studied subpopulations (see Appendix A, Table A5). Species richness ranged from 10 to 33 species in SP2 and SP6, respectively, indicating pronounced differences in local species pools along the Syr Darya river corridor.

Shannon diversity values ranged from 2.21 to 3.44, with the highest values recorded in SP6 and SP4 (3.07), whereas the lowest diversity occurred in SP2 (2.21). Simpson index values remained consistently high (0.88–0.97), indicating relatively even species distributions and the absence of strong monodominance within the studied communities.

Subpopulations located in the midstream section (SP5–SP6) generally exhibited higher species richness and structural diversity compared with most sites in the upstream section. However, the spatial distribution of α-diversity did not follow a strictly longitudinal pattern along the river. Instead, diversity patterns appear to reflect the combined influence of local environmental conditions, including habitat disturbance and anthropogenic pressure. Variation in community diversity may also be reflected in the demographic structure and regeneration patterns of *L. dasystemum* populations.

### 3.4. Floristic Similarity Among Subpopulations

Pairwise Sørensen similarity coefficients ranged from 12.5% to 51.9% (see Appendix A, Table A6), indicating generally low to moderate floristic similarity among the studied subpopulations.

The highest similarity was recorded between SP5 and SP6 (51.9%), both located in the midstream section of the river. In contrast, the lowest similarity was observed between SP1 and SP2 (12.5%), despite their geographical proximity.

Mean floristic similarity within the upstream section (SP1–SP4) was 24.9%, while the average similarity between upstream and midstream subpopulations was 23.5%. In contrast, similarity within the midstream section (SP5–SP6) was substantially higher (51.9%), indicating a more homogeneous community structure compared with the upstream sites, where floristic composition showed pronounced internal differentiation.

### 3.5. β-Diversity Partitioning

Partitioning of Sørensen dissimilarity into the components of species turnover (βsim) and nestedness (βsne) revealed that spatial differences among the studied subpopulations were primarily driven by species turnover rather than nestedness (see Appendix A, Table A7 and Table A8).

The turnover component (βsim) ranged from 33.3% to 81.0%. The highest values were observed between SP4 and SP5 (81.0%) and SP1 and SP2 (80.0%), indicating substantial differences in species composition between these communities. Comparisons between subpopulations located in the upstream and midstream sections also showed consistently high βsim values (≥58%), reflecting pronounced spatial differentiation along the river corridor.

In contrast, the nestedness component (βsne) was considerably lower, ranging from 0.6% to 26.7%. In most pairwise comparisons, its contribution did not exceed 12%, suggesting that simple species loss played only a minor role in shaping community dissimilarity.

### 3.6. Floristic Classification of Subpopulations

Floristic classification revealed clear differentiation of the six *L. dasystemum* subpopulations along the Syr Darya river corridor. In the upstream section (SP1–SP4), communities were mainly represented by Tugai associations of *Populetum pruinosae* in several subassociational variants as well as *Elaeagnetum angustifoliae*. In the midstream section (SP5–SP6), the syntaxonomic structure was simpler and included *Populetum pruinosae imperatetosum cylindricae* and *Tamaricetum hispidae* (see Appendix A, Table A9).

TWINSPAN classification identified two major floristic groups among the six subpopulations. The first division (eigenvalue = 0.693) separated the sites into Group I (SP2, SP3, SP5, SP6) and Group II (SP1, SP4) (see Appendix A, Table A10). These clusters differed in the composition of diagnostic species and the distribution of pseudospecies levels.

Group I was characterized by higher pseudospecies levels of *L. dasystemum* and the dominant component *Populus pruinosa*, together with the presence of *Phragmites australis*, *Cynodon dactylon*, *Fraxinus sogdiana*, *Artemisia ferganensis*, *Imperata cylindrica*, *Glycyrrhiza glabra*, and *Tamarix hispida*. The species composition reflects a mixture of floodplain and mesophytic elements, including several taxa tolerant of anthropogenic disturbance.

Group II (SP1 and SP4) was distinguished by a higher representation of *Elaeagnus angustifolia* and *Suaeda altissima*, indicating different habitat conditions and structural characteristics of the plant communities.

The cluster structure corresponds with the results of β-diversity partitioning, where the dominance of the turnover component (βsim) indicated species replacement among subpopulations. The clusters also broadly reflect spatial position along the river corridor and variation in anthropogenic disturbance levels. Subpopulations with higher disturbance, including SP3 (>25%) and SP2 and SP5 (10–25%), belonged to Group I together with SP6 (<5%), whereas SP1 (5–10%) and SP4 (<5%) formed Group II, representing sites with relatively lower habitat transformation.

### 3.7. Demographic Indicators and Population Age Structure

**Figure 4:**
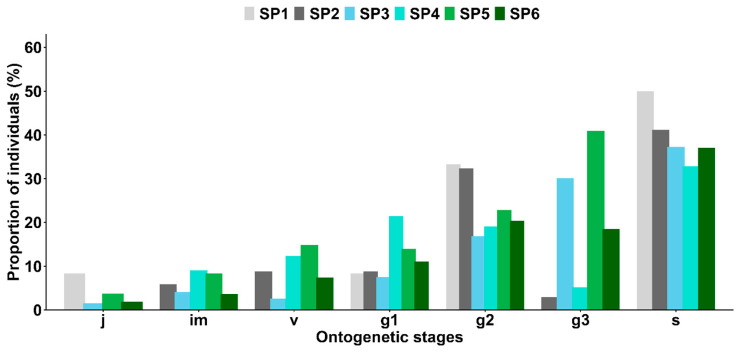
Ontogenetic structure of *Lycium dasystemum* subpopulations in the Syr Darya River floodplain. Bars represent the proportion of individuals (%) in different ontogenetic stages. Ontogenetic stages: j—juvenile; im—immature; v—virginal; g1—young generative; g2—mature generative; g3—old generative; s—senile.

Analysis of ontogenetic spectra revealed two main types of population structure among the studied subpopulations of *L. dasystemum*: left-skewed and centered age distributions (Figure 4).

Subpopulations SP1, SP4, and SP6 exhibited left-skewed ontogenetic spectra, characterized by a high proportion of virginile individuals (v), reaching 34.8–64.3%, together with the regular presence of juvenile and immature stages. The age index (Δ) in these subpopulations ranged from 0.18 to 0.34, while the efficiency index (ω) remained moderate, ranging from 0.53 to 0.62. The regeneration index (Iv) reached 0.90–2.50 (Table 2), indicating active recruitment and the presence of prereproductive individuals in the population structure.

Subpopulations SP3 and SP5 showed centered ontogenetic spectra, dominated by middle-aged generative individuals (g2), which accounted for 43.8–66.7% of all individuals. Prereproductive groups were present but comparatively less abundant. Values of Δ ranged from 0.36 to 0.41, while ω ranged from 0.76 to 0.83, corresponding to mature population states. Regeneration indices were lower than in the left-skewed subpopulations, with Iv values of 0.15 in SP3 and 0.50 in SP5. In both subpopulations, the replacement index (Iz) remained below 1.0, indicating that complete replacement of older individuals by younger cohorts was not achieved.

The most reduced regeneration was recorded in SP2, which occurred under moderately high anthropogenic disturbance. In this subpopulation, the ontogenetic spectrum was dominated by generative individuals, whereas juvenile and immature stages were absent or nearly absent. The regeneration index was minimal (Iv = 0), and ecological density was low (Pecol = 0.18 individuals m^−2^), indicating severely reduced recruitment and limited population renewal.

Overall, differences in population structure were broadly consistent with variation in local habitat conditions and disturbance intensity. Subpopulations occurring in relatively less transformed habitats maintained a higher proportion of prereproductive individuals, whereas disturbed sites were characterized by generative-dominated spectra, reduced regeneration, and incomplete generational replacement.

### 3.8. Vitality of Subpopulations

The assessment of vitality revealed clear differences in the functional condition of *L. dasystemum* subpopulations (Table 2). Based on the recalculated prosperity index (IQ), two subpopulations, SP1 and SP5, were characterized by IQ values above 1.0. In SP1, IQ reached 1.20, while the highest value was recorded in SP5 (IQ = 1.42). These subpopulations had a higher combined proportion of individuals belonging to the high and medium vitality classes than to the low vitality class, indicating a relatively favorable vitality structure.

The remaining subpopulations showed lower IQ values and were assigned to the range 0.5 ≤ IQ < 1. In SP2, SP3, SP4, and SP6, IQ varied from 0.50 to 0.85. SP2 and SP3 showed intermediate values of 0.85 and 0.81, respectively, whereas SP4 had a lower IQ value of 0.75. The lowest value was recorded in SP6 (IQ = 0.50), where the proportion of low-vitality individuals was comparatively high.

The IVC values ranged from 0.54 to 0.97 among the studied subpopulations. The lowest IVC value was observed in SP3 (0.54), while the highest values were recorded in SP4 (0.97), SP5 (0.93), and SP6 (0.90). However, the distribution of IVC and IQ values was not fully parallel. For example, SP4 and SP6 had relatively high IVC values but lower IQ values, indicating that higher mean morphometric performance does not necessarily correspond to a more favorable distribution of individuals among vitality classes.

**Figure A1:**
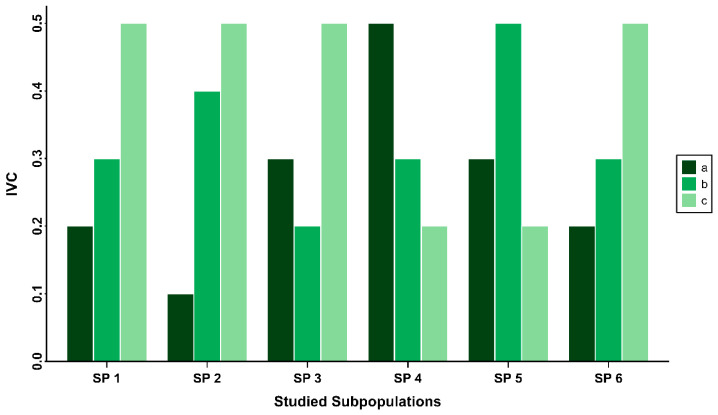
Vitality structure of *Lycium dasystemum* subpopulations. Bars represent the proportion of individuals belonging to different vitality classes. a—high vitality class; b—medium vitality class; c—low vitality class.

Overall, the vitality structure of *L. dasystemum* subpopulations was spatially heterogeneous (see Appendix A, Figure A1). SP5 showed the most favorable combination of IVC and IQ values, whereas SP3 was characterized by the lowest IVC and SP6 by the lowest IQ. These differences are interpreted descriptively and may reflect variation in local habitat conditions and disturbance intensity among the studied subpopulations (Table 3).

### 3.9. Morphological Variability and Phytocoenotic Plasticity

Morphometric analysis of *L. dasystemum* revealed differences in both intrapopulation variability (CV_mean_) and interpopulation plasticity (Ip) for the main biometric traits: plant height (H), shrub diameter (D), leaf width (Wh), and leaf length (Lfol) (Table 4).

Plant height varied from 116.0 cm (SP6) to 140.6 cm (SP1), with a mean intrapopulation variability of CV_mean_ = 12.6% and Ip = 17%. Among the analyzed traits, plant height showed the largest interpopulation range while maintaining moderate intrapopulation variability.

Shrub diameter ranged from 149.9 to 160.5 cm and exhibited lower variability (CV_mean_ = 9.2%) with moderate plasticity (Ip = 12%), indicating relative stability of this morphological trait across populations.

Leaf width demonstrated the highest plasticity (Ip = 20%) despite low intrapopulation variability (CV_mean_ = 7.36%). Mean values ranged from 2.24 to 2.95 cm, reflecting pronounced interpopulation differences while maintaining morphological consistency within subpopulations.

Leaf length varied between 6.27 and 6.96 cm and showed the lowest plasticity among the analyzed traits (Ip = 10%), with moderate variability (CV_mean_ = 11.2%). This indicates that leaf length varied less among subpopulations than plant height or leaf width. Therefore, leaf length appears to be a more conservative trait and less responsive to local habitat variation in the present dataset.

Intrapopulation variability of all traits remained within low to moderate levels (7.36–12.6%), whereas interpopulation differentiation was most pronounced for plant height and leaf width. A comparison of CV_mean_ and Ip values indicates that different morphological traits respond unequally to spatial heterogeneity of habitat conditions. The spatial organization of *L. dasystemum* populations may reflect the combined influence of floodplain hydrological conditions and anthropogenic pressure, which was expressed in differences in floristic composition, demographic structure, vitality parameters, and morphological variability among the studied subpopulations.

## 4. Discussion

The results show that Tugai vegetation along the Syr Darya River is structured by the combined influence of hydrological variability and anthropogenic disturbance, which together shape the demographic stability and regeneration patterns of *L. dasystemum* populations.

### 4.1. Hydrological Context and the Relevance of the Flood Pulse Concept

Floodplain forests are often interpreted within the framework of the Flood Pulse Concept, which emphasizes the ecological role of periodic flooding in maintaining the structure, productivity, and regeneration dynamics of riverine ecosystems [49,50]. In Tugai forests of Central Asia, seasonal flooding has historically contributed to habitat heterogeneity, supported the regeneration of key woody species such as *Populus pruinosa*, and influenced competitive relationships within floodplain plant communities [64,65,66].

In the present study, however, the Flood Pulse Concept was not tested as a formal hypothesis. The available hydrological data did not include a direct comparison with a reference period or site characterized by a significantly altered flood pulse. Therefore, this concept is used here only as a general ecological background for interpreting the role of seasonal hydrological variability in floodplain vegetation. The hydrological data showed a pronounced seasonal regime but no significant interannual trend during the observation period, indicating relatively stable hydrological variability at the interannual scale within the studied reach.

Despite seasonal hydrological fluctuations, the spatial differentiation of *L. dasystemum* subpopulations did not strictly correspond to their longitudinal position along the river channel. Although subpopulations from the midstream section showed slightly higher α-diversity values, the TWINSPAN classification and β-diversity partitioning indicated that compositional differences were not organized along a simple upstream–midstream hydrological gradient. These results suggest that local habitat heterogeneity and site-level disturbance may contribute to the observed vegetation mosaic.

Thus, the contemporary Tugai vegetation of the Syr Darya River should not be interpreted as an equilibrium system regulated solely by natural flood-pulse dynamics. Rather, the observed patterns appear to be associated with the combined influence of seasonal hydrological conditions, local habitat heterogeneity, and spatially uneven anthropogenic disturbance.

### 4.2. Species Turnover and Community Differentiation Under Disturbance

The predominance of the species turnover component (βsim) over the nestedness component (βsne) indicates that floristic differentiation among the studied communities was mainly associated with species replacement rather than with a sequential loss of species. However, because only six subpopulations were analyzed, this result should be regarded as exploratory [7,67].

The high βsim values observed among subpopulations, including some geographically proximate sites, suggest that community composition differs substantially among local habitats [68]. This pattern is consistent with the possible influence of local environmental filters, such as soil conditions, floodplain moisture availability, and anthropogenic disturbance. Nevertheless, the present dataset does not allow direct testing of the relative contribution of each factor.

The relatively low contribution of the nestedness component further indicates that the subpopulations do not represent simple subsets of one another along a degradation gradient. Instead, they appear to represent different local community configurations. These floristic differences provide an ecological context for interpreting the demographic and vitality variation observed among *L. dasystemum* subpopulations, but they should not be interpreted as definitive evidence of broad community reorganization without further replicated spatial and environmental analyses.

### 4.3. Metacommunity Approach and Spatial Mosaic

The discontinuous distribution of Tugai habitats along river corridors makes the metacommunity perspective useful as a conceptual framework. However, in the present study, metacommunity processes were not directly tested. The limited number of subpopulations and the absence of replicated spatial analyses restrict the strength of conclusions that can be drawn about species sorting, dispersal limitation, or other metacommunity mechanisms [69,70].

The TWINSPAN clustering of plant communities did not correspond strictly to the longitudinal position of subpopulations along the river. This indicates that the studied Tugai communities form a spatial mosaic rather than a simple linear gradient along the river channel. Such a pattern may be associated with local differences in habitat conditions and disturbance intensity.

Therefore, the observed floristic differentiation should be interpreted cautiously as evidence of compositional heterogeneity among the studied sites. Stronger conclusions about metacommunity organization would require a larger number of sampling sites, replicated plots, and direct analyses of spatial and environmental variables.

### 4.4. Hydrological Background and Local Disturbance Context

Over recent decades, Tugai forests of Central Asia have been influenced by hydrological regulation and increasing land-use pressure [71,72]. In the Syr Darya floodplain, hydrological conditions define the general abiotic background of floodplain habitats, while local anthropogenic disturbances may modify community composition and population parameters at the site scale.

The present results indicate that neither longitudinal position nor hydrological context alone fully explains the observed variability among *L. dasystemum* subpopulations. Differences in floristic composition, β-diversity, demographic structure, and vitality appear to be associated with a combination of seasonal hydrological conditions, local habitat heterogeneity, and anthropogenic pressure.

Therefore, the studied Tugai communities are best interpreted as a heterogeneous floodplain vegetation mosaic. This interpretation is consistent with the observed data, but it should be considered descriptive rather than a statistically confirmed causal model. Further studies with replicated plots and direct environmental measurements are needed to test the relative effects of hydrological variables and disturbance intensity more rigorously.

### 4.5. Demographic Structure as a Reflection of Environmental Gradients

The results indicate that the demographic condition of *L. dasystemum* subpopulations in the Syr Darya River valley is shaped by the combined influence of hydrological stability and anthropogenic disturbance. The occurrence of two ontogenetic spectrum types—left-skewed and centered—reflects different levels of population stability across spatially heterogeneous habitats. In plant population biology, such variation in age structure is widely regarded as a reliable indicator of population status and environmental conditions.

Left-skewed spectra (SP1, SP4, SP6), characterized by a high proportion of virginal individuals and the presence of all pregenerative stages, indicate active regeneration processes. Elevated recovery index values (Iv), together with moderate values of the age (Δ) and efficiency (ω) indices, suggest a balanced demographic structure and stable population renewal. Similar population structures are typical of species occurring in relatively stable floodplain habitats where regeneration conditions remain favorable [6].

In contrast, centered spectra (SP3, SP5) reflect demographic stabilization with reduced recruitment of younger individuals. The decline in pregenerative fractions and lower Iv values indicates slower regeneration processes. Comparable demographic shifts have been documented in plant populations experiencing increased ecological stress or altered habitat regimes [73].

The most vulnerable demographic state was observed in SP2 and partly in SP3, where intense anthropogenic pressure is associated with strong dominance of generative individuals (up to 87–100%) and an almost complete absence of juvenile plants. Low ecological density and minimal Iv values indicate weakened seed and vegetative regeneration. Such demographic configurations are commonly interpreted as signals of population degradation and elevated risk of local decline in disturbed environments [74,75].

Taken together, the age structure of the studied subpopulations acts as an integrative indicator of habitat conditions and reflects the influence of disturbance intensity and local environmental gradients.

### 4.6. Population Vitality and Functional Types of Stability

The vitality assessment provided additional information on the functional condition of *L. dasystemum* subpopulations and complemented the demographic analysis. In the present study, the prosperity index (IQ) and the index of vitality conditions (IVC) did not show identical patterns among subpopulations, indicating that population vitality should be interpreted using both the distribution of individuals among vitality classes and the mean morphometric condition of plants.

According to the prosperity index, SP1 and SP5 showed the most favorable vitality structure, with IQ values exceeding 1.0. In these subpopulations, the combined proportion of high- and medium-vitality individuals was greater than the proportion of low-vitality individuals. This suggests a relatively stable functional state and a more favorable balance among vitality classes. SP5 was especially notable because it combined a high IVC value with the highest IQ value, indicating both good morphometric performance and a favorable vitality-class structure. The combination of active vegetative renewal and a stable age structure provides these populations with a high adaptive potential, a pattern typical for plant populations functioning under near-optimal environmental conditions [75,76].

In contrast, SP2, SP3, SP4, and SP6 had IQ values within the range of 0.5 ≤ IQ < 1, indicating a reduced or intermediate vitality state. SP3 showed the lowest IVC value, suggesting weaker morphometric performance of individuals. SP6, despite having a relatively high IVC value, had the lowest IQ value because of the comparatively high proportion of low-vitality individuals. A similar discrepancy was observed in SP4, where the highest IVC value did not correspond to a favorable IQ value. These patterns show that high mean morphometric values alone do not necessarily indicate a stable vitality structure at the population level.

The divergence between IVC and IQ is important for interpreting the condition of *L. dasystemum* populations. IVC mainly reflects the relative morphometric development of individuals, whereas IQ reflects the balance between high-, medium-, and low-vitality classes. Therefore, subpopulations with high IVC but low IQ may contain well-developed individuals on average, while still having an unfavorable vitality-class distribution. Such cases may indicate internal heterogeneity of population condition and should be considered carefully in conservation assessments.

Overall, the vitality patterns of *L. dasystemum* subpopulations indicate spatial heterogeneity in functional condition across the studied Tugai habitats. These differences are consistent with variation in local habitat conditions and observed disturbance intensity, but they should be interpreted descriptively rather than as direct causal effects. The combined use of IVC and IQ provides a more balanced assessment of population vitality and helps identify subpopulations that may require closer monitoring, particularly those with low IQ values and a high proportion of low-vitality individuals.

The vitality and conservation status of plant species in Uzbekistan have been increasingly documented through regional IUCN assessments and floristic studies, underscoring the need for integrated population-level evaluations to inform conservation prioritization [77,78,79]. These vitality patterns support the demographic trends identified in the previous sections and demonstrate that population stability is determined not only by age structure but also by the functional condition of individuals. The joint analysis of demographic and vitality parameters therefore provides a more comprehensive assessment of population resilience under changing environmental conditions.

### 4.7. Morphological Plasticity as an Adaptive Mechanism

The relatively low to moderate level of phytocoenotic plasticity (Ip = 10–20%) indicates a limited ecological amplitude of *L. dasystemum*. Such restricted morphological flexibility suggests adaptation to floodplain habitats with a relatively stable moisture regime and is typical of species associated with specialised environments [80,81].

Leaf width exhibited the highest plasticity, reflecting a potential adaptive response to variability in water availability and light conditions. In moderately stressful environments, adjustments in leaf morphology may optimise photosynthetic efficiency and resource use [7].

In contrast, the relatively low plasticity of leaf length suggests a stronger genetic control of this trait. Plant height showed the highest variability (CV > 12.6%), indicating sensitivity to microhydrological conditions and anthropogenic disturbance. This trait may therefore serve as a useful bioindicator of habitat quality.

The observed morphological patterns indicate that *L. dasystemum* follows a moderately stress-tolerant strategy in which vegetative regeneration plays an important role. While such a strategy supports population persistence under short-term disturbances, it may limit adaptive capacity under prolonged alteration of floodplain hydrological regimes.

### 4.8. Integration of Demographic, Vitality, and Morphological Indicators

The integration of demographic, vitality, and morphological indicators showed that the condition of *L. dasystemum* subpopulations cannot be adequately described by a single index or a simple ranked sequence. The revised vitality assessment demonstrated that the index of vitality conditions (IVC) and the prosperity index (IQ) may show different patterns among subpopulations. Therefore, population condition was interpreted using a combination of demographic structure, regeneration indices, ecological density, IVC, IQ, and morphometric variability.

SP5 showed the most favorable combination of vitality indicators, with both a high IVC value and the highest IQ value. This suggests a comparatively stable functional state, although the site still requires attention because of the recorded level of anthropogenic disturbance. SP1 also had an IQ value above 1.0, indicating a favorable distribution of individuals among vitality classes, but its IVC value was lower than that of SP4–SP6. Thus, SP1 can be interpreted as functionally stable according to IQ, but not as the strongest subpopulation according to morphometric performance.

SP4 and SP6 illustrate the importance of using several indicators simultaneously. Both subpopulations had relatively high IVC values, suggesting good mean morphometric development of individuals. However, their IQ values were below 1.0, indicating a less favorable distribution of individuals among vitality classes. In SP6, this discrepancy was particularly evident because the subpopulation had the lowest IQ value despite a relatively high IVC. Such patterns indicate that high morphometric performance of some individuals does not necessarily correspond to a favorable vitality structure of the whole subpopulation.

SP2 and SP3 showed signs of reduced population condition according to several indicators. SP2 was characterized by weak regeneration and low ecological density, while SP3 combined the lowest IVC value with strong anthropogenic disturbance. These subpopulations therefore represent less favorable demographic and functional states in the present dataset.

Overall, the combined use of demographic, vitality, and morphological indicators provided a more balanced assessment of the *L. dasystemum* population condition than any single group of traits alone. The observed patterns should be interpreted cautiously because the study design was descriptive and included a limited number of subpopulations. Therefore, the differences among subpopulations are discussed as comparative and associative patterns rather than as statistically confirmed effects of hydrological or disturbance factors.

### 4.9. Ecological Implications, Uncertainty and Future Dynamics

The results indicate that subpopulations with weak regeneration, low ecological density, reduced vitality-class structure, or high disturbance pressure may be more vulnerable to further habitat degradation. Based on the combined demographic and vitality assessment, SP2 and SP3 should be considered priority subpopulations for conservation monitoring. SP2 showed limited recruitment, low ecological density, and a high proportion of generative individuals, indicating reduced population renewal. SP3 requires particular attention because it was associated with the highest trampling pressure, corresponding to stage V of degradation, and also had the lowest IVC value among the studied subpopulations.

SP6 should also be monitored, although its interpretation is more complex. This subpopulation had relatively high IVC and active recruitment, but the recalculated IQ value was the lowest among all subpopulations, indicating an unfavorable distribution of individuals among vitality classes. This suggests that monitoring should include not only recruitment and density but also the vitality-class structure of individuals. SP4 showed a similar, although less pronounced, discrepancy between high IVC and lower IQ; therefore, it should not be interpreted as uniformly favorable despite its strong morphometric performance.

SP5 had the most favorable combination of IVC and IQ values, but the recorded disturbance level indicates that it should be considered a secondary monitoring site to prevent future degradation. Maintaining its current functional state may require reducing local disturbance pressure, especially trampling and grazing, and preserving regeneration microsites.

From a management perspective, conservation measures should focus on reducing grazing pressure, trampling, cutting, and mechanical soil disturbance in the most vulnerable sites. Temporary protection of regeneration microsites, seasonal regulation of grazing, and repeated monitoring of recruitment, ecological density, IVC, IQ, and vitality-class distribution would provide practical tools for assessing future population dynamics. At the same time, maintaining sufficient floodplain moisture and ecological connectivity remains important for supporting the persistence of Tugai vegetation, although hydrology in this study should be interpreted as regional ecological background rather than as a directly tested driver of differences among subpopulations.

The interpretation of these results should consider the limitations of the study. Each subpopulation was represented by one main plot, and no independent replicate plots were established within subpopulations. In addition, site-specific hydrological measurements were not available. Therefore, the observed relationships between habitat conditions, disturbance, and population parameters should be treated as descriptive and comparative patterns rather than statistically confirmed causal effects. Future studies should include more subpopulations, replicated plots, repeated observations over time, and direct environmental measurements. Such data would allow correlation, regression, PERMANOVA, GLM/GLMM, and ordination methods to be applied for testing the relative effects of hydrological conditions and anthropogenic disturbance more rigorously.

## 5. Conclusions

This study suggests that *L. dasystemum* subpopulations in the Syr Darya River floodplain occur within a heterogeneous Tugai vegetation mosaic rather than following a simple upstream–midstream river gradient. The observed differences in floristic composition, β-diversity, demographic structure, vitality status, and morphological variability indicate spatial heterogeneity among the studied sites. However, these patterns should be interpreted as descriptive associations rather than as statistically confirmed causal effects of hydrological or disturbance factors.

The hydrological data showed pronounced seasonal variability and high synchrony between the upper and middle reaches of the Syr Darya River during 2020–2024. Therefore, hydrology was considered as a regional ecological background for interpreting the floodplain context, rather than as a directly tested driver of differences among individual subpopulations. Differences among subpopulations appear to be more closely associated with local habitat conditions, floodplain moisture context, and observed anthropogenic disturbance intensity.

The predominance of the species turnover component indicates that floristic differences among the studied Tugai communities were mainly related to species replacement rather than nestedness. This supports the interpretation of the study area as a spatially heterogeneous vegetation mosaic. At the population level, subpopulations differed in regeneration capacity, ecological density, vitality-class structure, and morphometric performance. SP5 showed the most favorable combination of IVC and IQ values, while SP1 also had an IQ value above 1.0. In contrast, SP2 and SP3 showed less favorable demographic and vitality-related characteristics, including weak regeneration, low ecological density, or reduced morphometric condition.

The revised vitality assessment showed that IVC and IQ provide complementary information and should not be interpreted as identical indicators. Some subpopulations, such as SP4 and SP6, had relatively high IVC values but lower IQ values, indicating that higher mean morphometric performance does not necessarily correspond to a favorable distribution of individuals among vitality classes. Therefore, conservation assessment of *L. dasystemum* should combine demographic indices, ecological density, IVC, IQ, and vitality-class distribution.

For practical conservation assessment, vulnerable subpopulations requiring priority management attention can be identified using the following operational thresholds: regeneration index below 0.5 (Iv < 0.5), replacement index below 1.0 (Iz < 1.0), ecological density below 0.20 individuals m^−2^ (Pecol < 0.20 individuals m^−2^), and vitality quality index below 1.0 (IQ < 1.0). These thresholds are used here as practical screening criteria rather than universal species-specific limits. According to these criteria, SP2 and SP3 require particular attention because of weak regeneration, while SP2 and SP5 fall below the ecological-density threshold. The replacement index remained below 1.0 in all studied subpopulations, indicating incomplete generational replacement across the study area. Based on the vitality quality index, SP2, SP3, SP4, and SP6 also require monitoring, whereas SP1 and SP5 showed a more favorable vitality-class structure.

From a practical perspective, SP2 and SP3 should be considered priority subpopulations for conservation monitoring. SP3 requires particular attention because it showed the highest trampling pressure, with more than 25% of the surface affected, corresponding to stage V of degradation. SP2 also requires monitoring because it combines weak recruitment, low ecological density, and dominance of generative individuals. SP6 should also be monitored because, despite relatively high IVC and active recruitment, it had the lowest recalculated IQ value, indicating an unfavorable vitality-class structure. SP5 can be considered a secondary monitoring site, where preventive grazing regulation may help avoid further degradation despite its favorable IQ value.

Feasible management actions include seasonal regulation of grazing, temporary protection of regeneration microsites, reduction in trampling, cutting, and other mechanical disturbances, and repeated monitoring of regeneration, ecological density, IVC, IQ, and vitality-class structure. Maintaining sufficient floodplain moisture, preventing further fragmentation of Tugai habitats, and preserving ecological connectivity among subpopulations remain important for supporting the long-term persistence of *L. dasystemum* and associated Tugai vegetation in Central Asia.

Future studies should include a larger number of subpopulations, replicated plots within subpopulations, repeated observations over time, and direct site-specific environmental and hydrological measurements. Such data would allow more rigorous testing of the relative roles of hydrological conditions, local habitat factors, and anthropogenic disturbance in shaping the population structure and vitality of *L. dasystemum*.

## Figures and Tables

**Table 1:** Weight coefficients used for calculating the age index $\left(\Delta \right)$ and efficiency index $\left(\omega \right)$ of cenopopulations.

Ontogenetic Stage	Weight Coefficient for Δ, K_i_	Efficiency Coefficient for ω, $e_{i}$
j	0.0	0.0
im	0.0	0.110
v	0.111	0.415
g1	0.257	0.779
g2	0.512	1.000
g3	0.726	0.768
s	0.947	0.283

**Table 2:** Demographic indices and age structure of *Lycium dasystemum* subpopulations.

SP	Δ	ω	D	P_ecol_	I_v_	I_s_	I_z_	Population Stage
^a^ SP1	0.34	0.60	0.23	0.38	0.90	0.1	0.43	maturing
^a^ SP2	0.28	0.80	0.11	0.18	0	0	0.00	maturing
^a^ SP3	0.41	0.83	0.13	0.32	0.15	0	0.13	mature
^a^ SP4	0.21	0.62	0.20	0.40	1.00	0	0.50	maturing
^b^ SP5	0.36	0.76	0.18	0.12	0.50	0	0.33	mature
^b^ SP6	0.18	0.53	0.28	0.23	2.50	0	0.21	young

Notes: ^a^—upper reach; ^b^—middle reach; Δ—age index; ω—efficiency index; D—population density; P_ecol_—ecological density; I_v_—regeneration index; I_s_—senescence index; I_z_—replacement index.

**Table 3:** Vitality indices and vitality types of *Lycium dasystemum* subpopulations.

SP	Vitality Class			IVC	IQ	Vitality Type
	a	b	c			
^a^ SP1	1.5	5.5	3.0	0.60	1.2	I_Q_ > 1
^a^ SP2	2.8	3.5	3.7	0.63	0.85	0.5 ≤ I_Q_ < 1
^a^ SP3	1.9	4.3	3.8	0.54	0.81	0.5 ≤ I_Q_ < 1
^a^ SP4	1.4	4.6	4.0	0.97	0.75	0.5 ≤ I_Q_ < 1
^b^ SP5	2.2	5.2	2.6	0.93	1.42	I_Q_ > 1
^b^ SP6	1.2	3.8	5.0	0.90	0.5	0.5 ≤ I_Q_ < 1

Note: ^a^—upper reach; ^b^—middle reach; IVC—index of vitality conditions; IQ—quality index.

**Table 4:** Morphological variability and phytocoenotic plasticity of *Lycium dasystemum*.

S	^a^ SP1	^a^ SP2	^a^ SP3	^a^ SP4	^b^ SP5	^b^ SP6	CV	I_p_
H	140.6 ± 3.7 8.4	135.8 ± 4.1 9.7	124.7 ± 7.7 19.4	118.8 ± 7.2 19.2	127.1 ± 7.4 18.4	116.0 ± 5.1 13.7	12.6	17
D	156.6 ± 7.5 15.1	156.7 ± 3.3 6.6	149.9 ± 3.9 8.3	160.5 ± 4.3 8.5	150.7 ± 3.9 8.4	160.5 ± 4.3 8.5	9.2	12
Wh	2.7 ± 0.1 7.4	2.9 ± 0.1 17.2	2.6 ± 0.1 8.2	2.8 ± 0.1 10.1	2.9 ± 0.1 10.4	2.2 ± 0.1 13.6	7.4	20
L_fol_	6.5 ± 0.1 4.8	6.9 ± 0.24 11.5	6.9 ± 0.2 9.3	6.6 ± 0.1 3.9	6.9 ± 0.2 10.3	6.3 ± 0.1 5.2	11.2	10

Notes: ^a^—upper reach; ^b^—middle reach; values are mean ± SE; H—plant height; D—shrub diameter; Wh—leaf width; L_fol_—leaf length; CV—coefficient of variation (%); I_p_—phytocoenotic plasticity index.

## Data Availability

Data will be made available on request. The datasets generated and analyzed during the current study are available from the corresponding author on reasonable request. Some data were obtained within the framework of the state program “Digital Nature” and the fundamental research project F-FA-2021-450; access to these datasets may be subject to institutional and legal restrictions.

## Appendix A

### Appendix A.1

**Table A1:** Environmental characteristics of *Lycium dasystemum* subpopulations in the Syr Darya River floodplain.

SP	Coordinates (N, E)	Plant Communities	Location	Anthropogenic Impact, %	Soil Type
SP1	40.802341° 70.964757°	Populetum pruinosae lycietosum dasystemi	upper reach	5.1–10	loam
SP2	40.785980° 71.052753°	Populetum pruinosae artemisietosum ferganensis	upper reach	10.1–25	salt-marsh
SP3	40.838854° 71.040138°	Populetum pruinosae zygophylletosum fabaginis	upper reach	>25	sandy-oam
SP4	40.881979° 71.449736°	Elaeagnetum angustifoliae suaedetosum altissimae	upper reach	5.1–10	loam
SP5	40.646728° 68.594514°	Populetum pruinosae imperatetosum cylindricae	middle reach	10.1–25	loam
SP6	40.987070° 68.616304°	Tamaricetum hispidae	middle reach	5.1–10	saline sand

### Appendix A.2

**Table A2:** Pairwise comparison of morphological traits among *Lycium dasystemum* subpopulations using Student’s *t*-test (α = 0.05).

SP	SP2	SP3	SP4	SP5	SP6
Height of the individual					
^a^ SP1	0.60 *	0.10 *	0.02 *	0.14 *	0.001
^a^ SP2		0.18 *	0.05 *	0.26 *	0.006
^a^ SP3			0.60 *	0.83 *	0.38 *
^a^ SP4				0.45 *	0.76 *
^b^ SP5					0.25 *
Woody shoot diameter					
^a^ SP1	0.1 *	0.07 *	0.08 *	0.42 *	0.08 *
^a^ SP2		0.68 *	0.59 *	0.20 *	0.59 *
^a^ SP3			0.83 *	0.14 *	0.83 *
^a^ SP4				0.16 *	1.00 *
^b^ SP5					0.16 *
Plant crown diameter					
^a^ SP1	0.99 *	0.46 *	0.67 *	0.51 *	0.67 *
^a^ SP2		0.22 *	0.51 *	0.28 *	0.51 *
^a^ SP3			0.10 *	0.89 *	0.10 *
^a^ SP4				0.13 *	1.00 *
^b^ SP5					0.13 *
Leaf width					
^a^ SP1	0.30 *	0.76 *	0.27 *	0.03	0.03
^a^ SP2		0.24 *	0.75 *	0.61 *	0.06 *
^a^ SP3			0.19 *	0.02	0.06 *
^a^ SP4				0.26 *	0.008
^b^ SP5					0.001
Leaf length					
^a^ SP1	0.14 *	0.16 *	0.16 *	0.10 *	0.12 *
^a^ SP2		0.79 *	0.79 *	0.97 *	0.03
^a^ SP3			1.00 *	0.75 *	0.02
^a^ SP4				0.75 *	0.02
^b^ SP5					0.02
Aboveground biomass					
^a^ SP1	0.09 *	0.02	0.17 *	0.02	0.17 *
^a^ SP2		0.47 *	0.07 *	0.22 *	0.07 *
^a^ SP3			0.22 *	0.67 *	0.22 *
^a^ SP4				0.27 *	1.00 *
^b^ SP5					0.27 *

Note: *—significant differences at *p* < 0.05. The communities are distributed in the upper (^a^) and middle (^b^) reaches of the Syr Darya River.

### Appendix A.3

**Table A3:** Descriptive statistics of hydrological values in the upper and middle reaches of the Syr Darya River (2020–2024).

River Reach	n	Mean	SD	Median	Min	Max	CV (%)
Upper Syr Darya	60	257.3	104.5	233.3	121.0	518.5	40.6
Middle Syr Darya	60	249.0	82.2	213.0	155.0	435.0	33.0

**Table A4:** Pearson correlation analysis between hydrological values of the upper and middle reaches of the Syr Darya River (2020–2024).

Method	Correlation Coefficient (r)	*p*-Value	95% CI (Lower)	95% CI (Upper)
Pearson correlation	0.951	<0.001	0.919	0.970

### Appendix A.4

**Table A5:** Alpha diversity indices (species richness, Shannon diversity, and Simpson index) across six subpopulations (SP1–SP6) located in the upper (^a^) and middle (^b^) reaches of the Syr Darya River.

SP	Richness	Shannon	Simpson
^a^ SP1	22	2.97	0.94
^a^ SP2	10	2.21	0.88
^a^ SP3	12	2.44	0.91
^a^ SP4	23	3.07	0.95
^b^ SP5	21	2.95	0.94
^b^ SP6	33	3.44	0.97

**Table A6:** Pairwise Sørensen similarity among six subpopulations (SP1–SP6), reflecting floristic resemblance derived from presence–absence data. The communities are distributed in the upper (^a^) and middle (^b^) reaches of the Syr Darya River.

SP	SP1	SP2	SP3	SP4	SP5	SP6
^a^ SP1	1					
^a^ SP2	12.5	1				
^a^ SP3	23.5	36.4	1			
^a^ SP4	35.6	18.2	22.9	1		
^b^ SP5	23.3	19.4	30.3	18.2	1	
^b^ SP6	18.2	23.3	26.7	28.6	51.9	1

**Table A7:** Pairwise turnover component of Sørensen dissimilarity (β_sim_) among six subpopulations (SP1–SP6) located in the upper (^a^) and middle (^b^) reaches of the Syr Darya River.

SP	SP1	SP2	SP3	SP4	SP5	SP6
^a^ SP1	0					
^a^ SP2	80.0	0				
^a^ SP3	66.7	60.0	0			
^a^ SP4	63.6	70.0	66.7	0		
^b^ SP5	76.2	70.0	58.3	81.0	0	
^b^ SP6	77.3	50.0	50.0	65.2	33.3	0.0

**Table A8:** Pairwise nestedness component of Sørensen dissimilarity (β_sne_) among six subpopulations (SP1–SP6) located in the upper (^a^) and middle (^b^) reaches of the Syr Darya River.

SP	SP1	SP2	SP3	SP4	SP5	SP6
^a^ SP1	0					
^a^ SP2	7.5	0				
^a^ SP3	9.8	3.6	0			
^a^ SP4	0.8	11.8	10.5	0		
^b^ SP5	0.6	10.6	11.4	0.9	0	
^b^ SP6	4.5	26.7	23.3	6.2	14.8	0

### Appendix A.5

**Table A9:** Characteristics of vegetation with the participation of Lycium dasystemum across different subpopulations (SP). SP1—Populetum pruinosae lycietosum dasystemi, SP2—Populetum pruinosae artemisietosum ferganensis, SP3—Populetum pruinosae, SP4—Elaeagnetum angustifoliae suaedetosum altissimae, SP5—Populetum pruinosae imperatetosum cylindricae, SP6—Tamaricetum hispidae species.

	LF	SP1 ^a^	SP2 ^a^	SP3 ^a^	SP4 ^a^	SP5 ^b^	SP6 ^b^
*Elaeagnus angustifolia* L.	T	2	–		3	–	–
*Fraxinus sogdiana* Bunge	T	–	–	1	1	1	1
*Populus pruinosa* Schrenk	T	4	2	2	–	3	–
*Amorpha fruticosa* L.	Sh	–	–	–	–	–	–
*Cynanchum acutum* L.	Sh	1	–	–	1	–	–
*Berberis heteropoda* Schrenk ex Fisch. & C.A.Mey.	Sh	1	–	–	–	–	–
*Halostachys caspica* (M.Bieb.) C.A.Mey.	Sh	–	1	–	–	–	1
*Caragana halodendron* (Pall.) Dum.Cours.	Sh	–	–	–	–	1	1
*Lycium dasystemum* Pojark.	Sh	3	2	1	2	1	1
*Punica granatum* L.	Sh	1	–	–	–	–	–
*Rosa canina* L.	Sh	1	–	–	–	–	–
*Rosa beggeriana* Schrenk ex Fisch. & C.A.Mey.	Sh	–	–	–	–	–	1
*Rubus* sp.	Sh	–	–	–	–	1	–
*Tamarix hispida* Willd.	Sh	–	2	1	–	–	2
*Tamarix ramosissima* Ledeb.	Sh	–	–	–	2	1	1
*Artemisia ferganensis* Krasch. ex Poljakov	SSh	–	3	–	–	–	–
*Artemisia subsalsa* Filatova	SSh	–	–	–	–	1	–
*Artemisia* sp.	P	–	–	–	–	–	1
*Aeluropus littoralis* (Gouan) Parl.	P	–	1	–	–	–	1
*Elymus repens* (L.) Gould	P	–	–	–	–	1	1
*Alhagi pseudalhagi* (M.Bieb.) Desv. ex Wangerin	P	–	–	–	–		1
*Asparagus persicus* Baker	P	1	–	1	–	1	1
*Clematis orientalis* L.	P	1	–	–	1	–	–
*Glycyrrhiza glabra* L.	P	–	–	–	–	1	1
*Cirsium arvense* (L.) Scop.	P	1	–	–	–	–	–
*Convolvulus arvensis* L.	P	1	–	–	–	1	1
*Corydalis repens* Mandl & Muhldorf	P	1	–	–	–	–	–
*Cynodon dactylon* (L.) Pers.	P	–	–	–	1	–	3
*Dodartia orientalis* L.	P	–	–	–	–	–	1
*Equisetum ramosissimum* Desf.	P	1	–	–	–	1	1
*Tripidium ravennae* (L.) H.Scholz	P	–	–	–	–	2	–
*Imperata cylindrica* (L.) Raeusch.	P	–	–	–	–	3	–
*Juncus bufonius* L.	P	–	–	–	–	–	1
*Lactuca tatarica* (L.) C.A.Mey.	P	1	–	–	–	–	1
*Lepidium latifolium* L.	P	–	–	–	1	–	1
*Leymus multicaulis* (Kar. & Kir.) Tzvelev	P	–	–	1	–	–	–
*Limonium otolepis* (Schrenk) Kuntze	P	–	1	–	–	–	–
*Poa bulbosa* L.	P	–	–	–	–	–	1
*Polypogon fugax* Nees ex Steud.	P	–	–	–	–	–	1
*Phragmites australis* (Cav.) Trin. ex Steud.	P	–	1	1	1	2	2
*Rumex dentatus* L.	P	–	–	–	1	–	–
*Saussurea salsa* (Pall.) Spreng.	P	–	–	–	–	–	1
*Sophora alopecuroides* L.	P	–	–	1	–	–	–
*Taraxacum comitans* Kovalevsk.	P	1	–	–	–	–	–
*Trifolium repens* L.	P	–	–	–	1	–	–
*Zygophyllum fabago* L.	P	–	–	2	–	–	–
*Onopordum acanthium* L.	A	–	–	–	1	–	–
*Artemisia annua* L.	A	–	–	–	1	–	1
*Bromus oxyodon* Schrenk	A	1	–	–	–	–	–
*Bromus scoparius* L.	A	–	–	–	1	–	1
*Cannabis sativa* L.	A	1	–	–	1	–	–
*Carduus pycnocephalus* L.	A	2	–	–	–	–	–
*Chenopodium album* L.	A	–	–	–	1	–	–
*Climacoptera lanata* (Pall.) Botsch.	A	–	–	1	–	–	–
*Crepis pulchra* L.	A	–	–	–	–	–	1
*Diarthron vesiculosum* (Fisch. & C.A.Mey. ex Kar. & Kir.) C.A.Mey.	A	–	–	–	–	1	–
*Eremopyrum triticeum* (Gaertn.) Nevski	A	–	1	–	–	–	–
*Euphorbia helioscopia* L.	A	–	–	1			1
*Euphorbia lamprocarpa* (Prokh.) Prokh.	A	–	–	–	–	–	–
*Galium spurium* L.	A	1	–	–	1	–	–
*Hordeum murinum* L.	A	1	–	1	1	–	–
*Medicago minima* (L.) Bartal.	A	–	–	–	–	1	1
*Potentilla reptans* L.	A	–	–	–	1	–	–
*Plantago major* L.	A	1	–	–	–	–	–
*Phleum paniculatum* Huds.	A	–	–	–	–	–	1
*Polygonum aviculare* L.	A	–	–	–	1	–	–
*Silene conica* L.	A	–	–	–	–	1	1
*Sisymbrium altissimum* L.	A	1	–	–	–	–	–
*Suaeda altissima* (L.) Pall.	A	–	1		2		
*Torilis arvensis* (Huds.) Link	A	–	–	–	–	1	–
*Verbascum blattaria* L.	A	–	–	–	–	–	–
*Vulpia* sp.	A	–	–	–	–	2	2
*Xanthium strumarium* L.	A	–	–	–	–	1	1

Note: *The communities are distributed in the upper (^a^) and middle (^b^) reaches of the Syr Darya River*.

### Appendix A.6

**Table A10:** TWINSPAN-derived floristic differentiation of six *Lycium dasystemum* subpopulations (SP1–SP6) along the Syr Darya River, indicating pseudospecies levels of diagnostic taxa within the two primary clusters. The communities are distributed in the upper (^a^) and middle (^b^) reaches of the Syr Darya River.

	0	0	0	0	1	1	
111	1	∙	∙	∙	∙	4	*Suaeda altissima*
111	∙	∙	∙	∙	4	5	*Elaeagnus angustifolia*
110	3	1	1	1	5	2	*Lycium dasystemum*
01	5	5	5	∙	5		*Populus pruinosa*
001	1	1	3	3	∙	1	*Phragmites australis*
001	∙	∙	∙	5	∙	1	*Cynodon dactylon*
001	∙	1	2	2	∙	1	*Fraxinus sogdiana*
000	∙	∙	3	3	∙	∙	*Vulpia* sp.
000	∙	∙	5	∙	∙	∙	*Imperata cylindrica*
000	∙	∙	2	5	∙	∙	*Glycyrrhiza glabra*
000	5	∙	∙	∙	∙	∙	*Artemisia ferganensis*
000	3	1	∙	4	∙	∙	*Tamarix hispida*
	^a^ SP2	^a^ SP3	^b^ SP5	^b^ SP6	^a^ SP1	^a^ SP4	

Note: The gray background is used only to facilitate the visual interpretation of the TWINSPAN classification by highlighting the distribution pattern of diagnostic taxa across subpopulations. The background shading has no statistical significance. The symbol “∙” indicates the absence of a species in the corresponding subpopulation (i.e., no recorded occurrence).
